# Role of anatomical pathways in shaping posterior alpha oscillations in the resting human brain

**DOI:** 10.1186/1471-2202-14-S1-P98

**Published:** 2013-07-08

**Authors:** R Hindriks, M Woolrich, M Kringelbach, H Luckhoo, M Joensson, H Mohseni, G Deco

**Affiliations:** 1 Center for Brain and Cognition, Computational Neuroscience Group, Department of Information and Communication Technologies, Universitat Pompeu Fabra, Barcelona, 08018, Spain; 2 Oxford Centre for Human Brain Activity, University of Oxford, Warneford Hospital, Oxford OX37JX, UK; 3 Department of Psychiatry, University of Oxford, Oxford, UK; 4 Center of Functionally Integrative Neuroscience (CFIN), Aarhus University, Denmark; 5 Centre for Doctoral Training in Healthcare Innovation, Institute of Biomedical Engineering, Department of Engineering Science, University of Oxford, UK

## 

Since their discovery almost a century ago, ongoing alpha oscillations as recorded with electroencephalography (EEG) or magnetoencephalography (MEG) have been associated with numerous mental and emotional states and have been hypothesized to play a crucial role in perceptual and cognitive processing [1]. A prominent feature of alpha oscillations recorded in the absence of stimuli or explicit tasks is their dominance over parietal-occipital midline regions [2]. In this study we combine MEG and diffusion spectrum imaging (DSI) to investigate the extent to which the topology of anatomical pathways can explain this dominance. We found that source-projected MEG alpha power correlates with eigenvalue centrality of the DSI-derived structural matrix [3]. In particular, the occipital-parietal dominance could largely be explained by the high density of structural connections within the posterior-medial parts of the structural core [4]. Moreover, more local network characterizations such as clustering coefficient, degree, and node centrality, were unable to explain the posterior dominance, suggesting that alpha power is shaped by global rather than local structural features.

To assess the possibility of a causal link between the DSI-derived structural network and the power topography of resting-state alpha oscillations, we constructed a computational model of large-scale brain dynamics. Within the model, alpha oscillations are generated within local circuits [5] and interact through long-range excitatory projections according to the DSI-derived structural topology. We found that, when structurally connected, alpha oscillations indeed dominate over parietal-occipital midline regions. Furthermore, they only did so when the dynamics was in the vicinity of an instability, which is in line with previous modeling work on resting-state BOLD correlations [5]. These findings suggest that the posterior dominance of alpha oscillations could indeed be shaped by the topology of anatomical pathways and that critical dynamics are required. We subsequently investigated which features of the experimentally identified network were crucial in shaping the observed dominance and assessed the role of coherent oscillations. In sum, this study provides experimental and theoretical evidence that alpha oscillations in the human resting brain are structured by the topology of underlying anatomical pathways.
